# A computed tomographic evaluation of femoral and tibial rotational reference axes in total knee arthroplasty

**DOI:** 10.1051/sicotj/2023002

**Published:** 2023-01-27

**Authors:** Johncy Panicker, Jai Thilak

**Affiliations:** 1 Amrita Institute of Medical Sciences Kochi Kerala 682041 India; 2 Clinical Professor in Orthpaedics, Amrita Institute of Medical Sciences Kochi Kerala 682041 India

**Keywords:** Rotational alignment, Rotational component positioning, Reference axes, Trans epicondylar axis, Osteoarthritis, Arthroplasty

## Abstract

*Introduction:* The surgical trans epicondylar axis (sTEA) is considered the gold standard for optimum rotation of the femoral component; however, no consensus exists on tibial component positioning. The objectives of this study were to determine the relationship of sTEA to various femoral and tibial reference axes in varus osteoarthritis (OA) knees and (ii) to study the intra-observer and inter-observer variability of the axis relationships. *Materials and methods:* The study was done on preoperative computerised tomogram (CT) scans of 110 varus knees to assess the rotational relationships respectively of femoral side sTEA with whitesides line (WSL), posterior condylar axis (PCA), clinical trans epicondylar axis (cTEA) and on the tibial side sTEA with posterior tibial margin (PTM), anterior condylar axis (ACA), Akagi’s line and line from the geometric centre of the tibial plateau to 1/3rd tibial tubercle (line GC 1/3rd TT). *Results:* On the femoral side the mean angles of sTEA with WSL, PCA, cTEA were 95.64° ± 2.85°, 1.77° ± 1.88°, 4.19° ± 0.99° respectively. On the tibial side, the mean angles of sTEA with, PTM, ACA, Akagi’s line, and line GC 1/3rd TT were 1.10° ± 4.69°, 11.98° ± 4.51°, 2.43° ± 4.35°, 16.04° ± 5.93° respectively. *Conclusion:* Contrary to the generalization, TEA has variable relationships. The surgical trans epicondylar axis was not at the assumed 3° of external rotation to PCA in 85% of knees, nor perpendicular to WSL in >95% of knees. Of the four tibial axes, Akagi’s line was the least variable with sTEA. Furthermore, surgeons should also be aware of the multiple reference axes and the range of deviation from sTEA to optimize the rotational alignment of components.

## Introduction

In total knee arthroplasty (TKA), attaining a balanced knee by optimising the femoral and tibial component rotational alignment is critical for functional outcomes and implant longevity [1–5]. An unbalanced knee can lead to instability, patellofemoral maltracking, persistent knee pain, stiffness, premature polyethylene wear, and unfavourable outcomes [1–3, 6]. According to the literature, rotational alignment of the tibial baseplate in TKA has garnered remarkably low attention and can be difficult due to the surgeon’s desire to maximise tibial bone coverage while achieving optimal component rotational alignment [1–6]. Although externally rotating the tibial component moves the tibial tubercle internally, thereby improving patellar tracking, excessive external rotation likely causes posterolateral suspension of the tibial baseplate and aggravation of the tibial internal torsion, resulting in in-toeing gait in patients with varus deformity of the knee [7, 8].

In primary TKA, on the femoral side, three commonly used landmarks for referencing rotational alignment of femoral component are the posterior condylar axis (PCA), the line connecting the posterior-most points of medial and lateral femoral condyles, plus a few degrees of fixed external rotation [9] (3° most common), Whiteside’s line (WSL), the line between the deepest points of trochlear groove anteriorly and intercondylar notch posteriorly [10] and trans epicondylar axes (TEA), “surgical” TEA (sTEA) and “clinical” TEA (cTEA) [11–15]. The “sTEA” is a line connecting the central sulcus of the medial epicondyle and the lateral epicondylar prominence, whereas “cTEA” also known as anatomic TEA, connects the most prominent and easily palpable points of the medial and lateral epicondyles. As evidenced by several studies, each of these techniques has its own array of advantages and pitfalls, with significant variability in the relationships between these axes among different ethnicities [10, 12–14, 16]. The sTEA is regarded as the “gold standard” for rotational alignment of both the femoral and tibial components to achieve a well-balanced TKA, presumably because it approximates the flexion-extension axis of the knee and the origin of femoral collateral ligaments [11]; however, it is challenging to project onto the tibial plateau intraoperatively. Therefore, several techniques exist for determining tibial rotational alignment on the tibial side, with no sufficient consensus regarding the most reliable tibial reference axes compared to the femur.

Some of the known examples of tibial axes are the posterior tibial margin (PTM) [8], Akagi’s line connecting the centre of the posterior cruciate ligament insertion to the medial edge of patellar tendon attachment [17], the axis using the geometric centre of the resected tibial plateau and medial third of patellar tendon attachment defined by Uehara et al. [18], anatomical tibial axis as defined by Cobb et al. [19] the axis going from 1 mm medial of the tibial tubercle medial edge to between the midsulcus of the tibial spines as defined by Dalury et al. [20].

The rationale for this study is for comparing the reference axes of both the distal femur and proximal tibia with controversial aspects regarding the same because of the limited studies available. Furthermore, previous studies have predominantly evaluated the relationship of TEA with other femoral axes, and tibial reference axes are evaluated to a lesser extent. The purpose of this study was to determine the relationship of sTEA to various femoral and tibial references axes in OA knees with varus deformity and to investigate whether the assumed classical rotational relationships of sTEA to both WSL and PCA of femur hold true and the relationship of sTEA to the axis of the tibia.

## Materials and methods

IRB-approval and written informed consent were obtained before commencing our study of 73 consecutive patients undergoing robotic-arm-assisted knee arthroplasty (both unilateral and bilateral). Our inclusion criteria were patients with varus deformity only between 0° and 20°, fixed flexion deformity (FFD < 10°), Type 1 sulcus “clearly visible” and Type 2 sulcus “difficult to recognise” according to Akagi et al. [21, 22]. We excluded patients with inflammatory arthritis, secondary OA following intraarticular fractures, varus deformity (>20°) and valgus knees, FFD > 10° (vi) unidentifiable medial sulcus on axial CT section [21, 22].

A standard radiological evaluation was followed, which included:

- Anteroposterior long-leg standing scanogram to determine the severity of varus deformity of the lower limb.
- Preoperative CT scan for image-based robotic-arm assisted arthroplasty of the arthritic knee in a supine position. All scans were performed on an institution CT scan system (Philips, USA), which included 5 mm slices through the hip and ankle joints and 1 mm slices through the knee joint. For CT imaging, a leg holder was used to hold the lower extremity in a stable rotational position with maximum extension.

**Figure 1:**
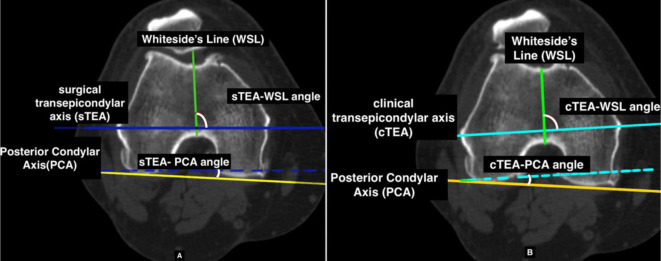
Angles measured on the distal femur in relation to (a) sTEA and (b) cTEA.

Two independent observers evaluated the scanogram and preoperative axial CT images of the knee twice, and a mean of the measurements was taken to minimise inter-and intra-observer variability. A sequence of landmark identification and analysis was adopted in each case to ensure consistency. First, the relevant landmarks of the distal femur with corresponding axes were marked on an axial CT image at the level of the epicondyles, where the medial sulcus was most clearly identified, to analyse the following as given in Figure 1:

1. “sTEA–PCA” angle formed by the sTEA and PCA
2. “cTEA–PCA” angle formed by the cTEA and PCA
3. “sTEA–WSL” angle formed by the sTEA and WSL
4. “cTEA–WSL” angle formed by the cTEA and WSL
5. “cTEA–sTEA” angle between the cTEA and sTEA

**Figure 2:**
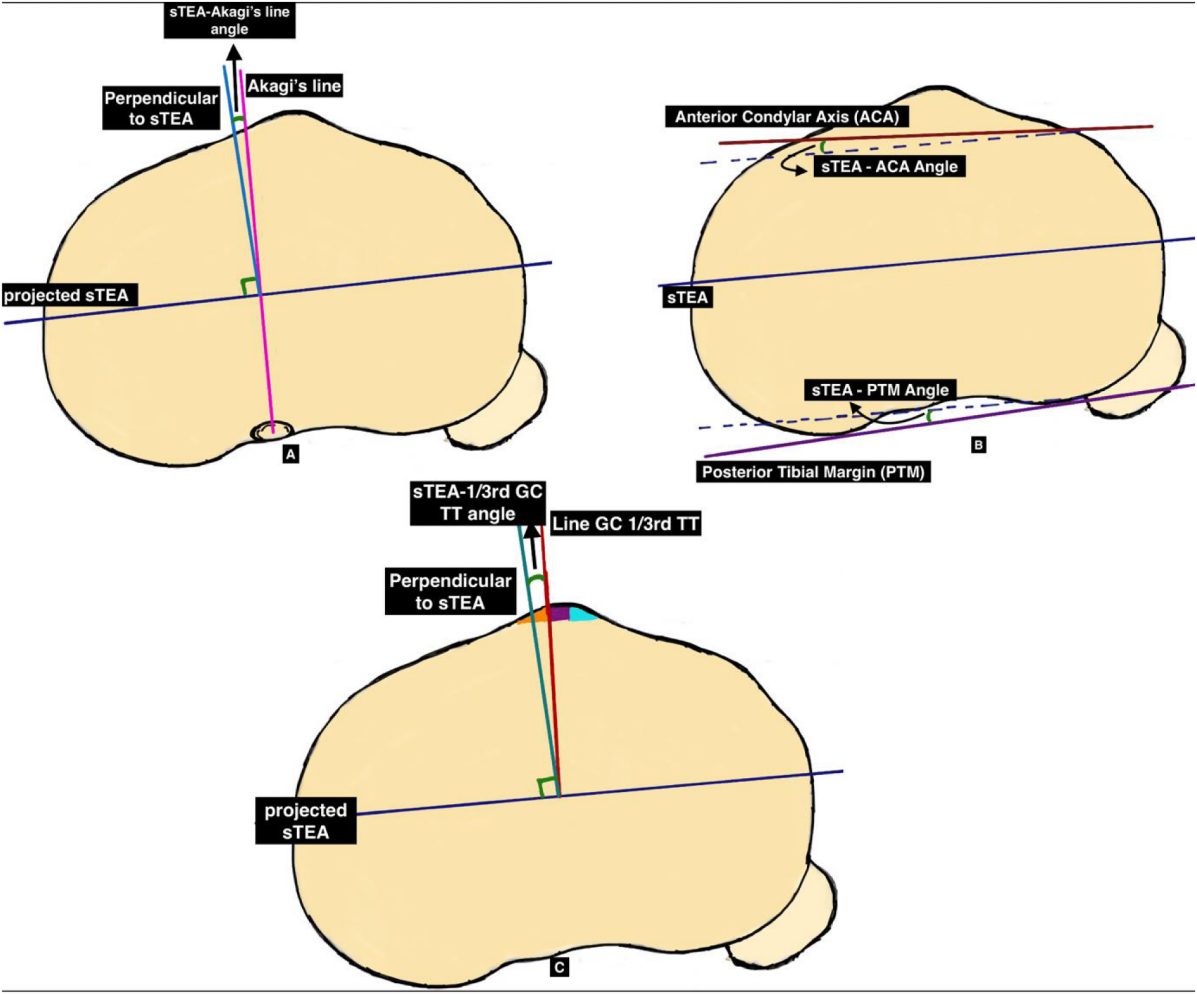
Angles measured on the proximal tibia in relation with sTEA, (A)’sTEA–Akagi’s line”, (B) “sTEA–ACA” and “sTEA-PTM” (C)’sTEA–line GC 1/3rd TT’.

Furthermore, two images of the proximal tibia at the level of the optimal osteotomy (8–10 mm below the highest point of the lateral plateau) and at the level of patellar tendon attachment were selected; on which the sTEA was projected on the axial CT tibial images to measure the relationship of sTEA with posterior tibial margin (PTM), anterior condylar axis (ACA), is the axis passing through the most anterior points of the lateral and medial of the tibial condyle, Akagi’s line and line from the geometric centre of the tibial plateau to the junction of medial 1/3rd and lateral 2/3rd tibial tubercle (line GC 1/3rd TT) as shown in Figure 2:

1. “sTEA–Akagi’s line” angle formed perpendicular to sTEA and Akagi’s line.
2. “sTEA–PTM” angle by sTEA and PTM (axis through most posterior points of tibial condyles).
3. “sTEA–ACA” angle by sTEA and ACA (axis through the most anterior points of medial and lateral condyles of the tibia).
4. “sTEA–line GC 1/3rd TT” angle formed by the perpendicular to sTEA and line GC 1/3rd TT’.

A positive value recorded outward from the midline was considered external rotation (ER), and a negative value noted inward as internal rotation (IR). Demographic details, the severity of varus deformity, and the rotational relationships were recorded in an Excel spreadsheet (Microsoft Corporation, USA).

Statistical analysis was performed using IBM SPSS version 20.0 software. Categorical variables were expressed using frequency and percentage, whereas numerical variables were presented using mean and standard deviation. Intra-observer and inter-observer reliability between 2 observers at six weeks intervals, intra-class correlation (ICC) were used with excellent being 0.75–1.00. Pearson’s correlation coefficient was used to study the relationship between parameters. A *p*-value of <0.05 was considered to be statistically significant.

## Results

The study includes 73 patients (59 females, 14 males), 42–84 years old, with a mean age of 63.51 ± 8.92 years. Thirty-seven underwent bilateral and 36 (24 right and 12 left) unilateral robotic-arm assisted knee arthroplasty, accounting for 110 knees (*n* = 110) as given in Table 1. The gender difference in the data was not looked into since the majority of our patients were females.

**Table 1:** Demographic data.

Parameters		(*n* = 73)	Percentage (%)
Age (years)	<65	34	47
	≥65	39	53
Gender	Male	14	19
	Female	59	81
Side	Right	24	33
	Left	12	16
	Bilateral	37	51
Body mass index (BMI in kg/m^2^)	>18.5–24.9 kg/m^2^ (normal)	15	20.54
	25–29.9 kg/m^2^ (pre-obesity)	36	49.31
	30.0–34.9 kg/m^2^ (class I obesity)	15	20.54
	35.0–39.9 kg/m^2^ (class II obesity)	7	9.59
Varus (*n* = 110)	<10	48	44
	≥10	62	56
Sulcus (*n* = 110)	Type 1 (distinctly visible sulcus)	80	72.73
	Type 2 (recognizable with difficulty)	30	27.27

On the femoral side (Table 2), we noted the sTEA was at a mean of 1.77° ± 1.88°, ranging from 2.65° of internal rotation to 7.29° of external rotation to PCA. The cTEA was found to be ranging from 1.23° to 10.33° with a mean external rotation of 5.91° ± 1.82° of external rotation to PCA. The mean angle between cTEA and sTEA was 4.19°, ranging between 1° and 7°. The angle formed by WSL to sTEA and the cTEA showed a mean external rotation of 95.64° ± 2.85° (range, 87.68–101.64) and 91.31° ± 2.78° (range, 84.44–97.11) respectively.

**Table 2:** Rotational relationships of sTEA with other axes on the femur and tibia.

Measurements on the femur (°)	
sTEA–PCA angle formed by the sTEA and PCA	Mean = 1.77 ± 1.88 (range = −2.65 to 7.29)
cTEA–PCA angle formed by the cTEA and PCA	Mean = 5.91 ± 1.82 (range = 1.23 to 10.33)
sTEA–WSL angle formed by the sTEA and WSL	Mean = 95.64 ± 2.85 (range = 87.68 to 101.64)
cTEA–WSL angle formed by the cTEA and WSL	Mean = 91.31 ± 2.78 (range = 84.44 to 97.11)
cTEA–sTEA angle between the cTEA and sTEA	Mean = 4.19 ± 0.99 (range = 0.77 to 7.19)
Measurements on the tibia (°)	
sTEA–Akagi’s line angle, formed by perpendicular to sTEA and Akagi’s line	Mean = 2.43 ± 4.35 (range = −8.68 to 14.84)
sTEA–PTM angle, formed by sTEA and the PTM	Mean = 1.10 ± 4.69 (range = −5.81 to 13.88)
sTEA–ACA angle, formed by sTEA and the ACA	Mean = 11.98 ± 4.51 (range = 1.47 to 22.92)
sTEA–line GC 1/3rd TT angle, formed by perpendicular to sTEA and line GC 1/3rd tibial tubercle	Mean = 16.04 ± 5.93 (range = −1.07 to 34.19)

In only 16 (14.5%) knees, sTEA was at an assumed 3° of external rotation to PCA. 85.5% of knees, i.e., 74 (67.27%) knees, were at less than 3° external rotation, and the remaining 20 (18.18%) knees had more than 3° of external rotation of sTEA. It was also noted that in only 7 (6.4%) knees, cTEA was at assumed 3° external rotation to PCA while 99 (90.18%) knees had more than 3° as given in Table 3.

**Table 3:** Proportion of patients who vary from the assumed PCA to TEA relationship.

Assumed classical relationships	sTEA vs PCA	cTEA vs PCA
	3° external rotation	3° external rotation
At zero	9	–
1°	23	1
Internally rotated	9	–
2°	28	3
Internally rotated	3	–
3°	16	7
Internally rotated	2	–
4°	14	12
≥5°	6	87
Total (*n*)	110	110
Inference	16/110 (14.5%) knees, sTEA was at 3° external rotation to PCA, 74/110 (67.27%) knees were at less than 3° external rotation	7/110 (6.4%) knees, cTEA was at 3° external rotation to PCA

In only 3 of 110 (2.73%) knees, WSL was found perpendicular to sTEA, while 71 of 110 (64.55%) knees was noted ≥5° deviation, and in 10 of 110 (9.1%) knees, WSL was perpendicular to cTEA. In 28 of 110 (25.45%), WSL was at 1° deviation either externally or internally to cTEA, which was the most common observation. Table 4.

**Table 4:** Proportion of patients who vary from the assumed WSL to TEA relationship.

Assumed classical relationships	WSL vs sTEA	WSL vs cTEA
	Perpendicular	Perpendicular
Normal	3	10
1°	4	28
Internally rotated	–	9/28
2°	9	24
Internally rotated	2/9	8/24
3°	13	21
Internally rotated	–	5/21
4°	10	10
Internally rotated	–	2/10
≥5°	71	17
Internally rotated	–	4/17
Total (*n*)	110	110
Inference	3/110 (2.73%) knees, WSL perpendicular to sTEA, 71/110 (64.55%) ≥5° deviation was noted	10/110 (9.1%) knees, WSL perpendicular to cTEA, 28/110 (25.45%) was at 1° deviation

**Table 5:** Comparison of tibial reference axes relationships with sTEA.

	Akagi’s line	ACA	PTM	Line GC-1/3rd TT
Mean	2.43	11.98	1.1	16.04
Standard deviation	4.35	4.5	4.69	5.93
Range	23.52	21.45	19.7	35.26
Minimum	−8.68	1.47	−5.81	−1.07
Maximum	14.84	22.92	13.88	34.19

**Table 6:** Intraobserver reliability among different axis relationships.

Axes relationships	*n*	Correlation (*r*)	*p* value
sTEA–PCA observer 1	110	0.82	<0.001
sTEA–PCA observer 2	110	0.856	<0.001
sTEA–WSL observer 1	110	0.79	<0.001
sTEA–WSL observer 2	110	0.798	<0.001
cTEA–PCA observer 1	110	0.81	<0.001
cTEA–PCA observer 2	110	0.873	<0.001
cTEA–WSL observer 1	110	0.75	<0.001
cTEA–WSL observer 2	110	0.784	<0.001
sTEA–Akagi’s line observer 1	110	0.88	<0.001
sTEA–Akagi’s line observer 2	110	0.899	<0.001
sTEA–PTM observer 1	110	0.97	<0.001
sTEA–PTM observer 2	110	0.965	<0.001
sTEA–line GC 1/3rd TT observer 1	110	0.85	<0.001
sTEA–line GC 1/3rd TT observer 2	110	0.878	<0.001
sTEA–ACA observer 1	110	0.91	<0.001
sTEA–ACA observer 2	110	0.926	<0.001

**Table 7:** Interobserver reliability among different axis relationships.

Intraclass correlation		95% Confidence interval		*F* test with true value 0			
		Lower bound	Upper bound	Value	df1	df2	*p* value
sTEA–PCA	0.899	0.852	0.931	9.876	108	108	<0.001
sTEA–WSL	0.886	0.834	0.922	8.777	109	109	<0.001
cTEA–PCA	0.894	0.845	0.927	9.412	109	109	<0.001
cTEA–WSL	0.874	0.816	0.913	7.925	109	109	<0.001
sTEA–Akagi’s line	0.938	0.909	0.957	16.112	109	109	<0.001
sTEA–PTM	0.943	0.938	0.97	34.287	109	109	<0.001
sTEA–line GC 1/3rd TT	0.919	0.882	0.944	12.339	109	109	<0.001
sTEA–ACA	0.95	0.927	0.966	19.965	109	109	<0.001

On the tibial side as shown in Tables 2 and 5; the mean of the angles formed by the perpendicular to sTEA with Akagi’s line, was 2.43° ± 4.35°, ranging from 8.68° of internal rotation to 14.84° of external rotation and with line GC 1/3rd TT, was 16.04° ± 5.93° ranging from −1.07 of internal rotation to 34.19° of external rotation respectively. The mean values of the angles formed by the sTEA with PTM were 1.10° ± 4.69° (range, −5.81° to 13.88°) external rotation, and with ACA was 11.98° ± 4.51°, (range, 1.47–22.92) of external rotation.

The intraclass correlation coefficients for the angles were evaluated for intraobserver and interobserver reliability respectively, as given in Tables 6 and 7. The relationship of sTEA with PCA had the best intraobserver and interobserver reliability among the femoral axes while among the tibial axes, PTM had the best intraobserver reliability and line GC 1/3rd TT had the least interobserver reliability.

## Discussion

To our knowledge, this is among the few studies to determine rotational relationships between reference axes of both femur and tibia in OA knees with varus deformity. Almost all of the contemporary posterior referencing jig-based TKA systems strongly favour a 3° external rotation to the PCA to consistently set the femoral component to attempt and establish a rectangular flexion gap [23]. This is usually rational in a knee with normal posterior condyle anatomy, where an external rotation of 3° can re-establish the flexion gap parallel to the resected tibial surface [24].

In our study, the sTEA was externally rotated 1.77° to the PCA with a range of 10°. The WSL was noted externally rotated at an average of 95.64° to the sTEA with a 14° range. The cTEA–PCA always obtained a positive value, whereas the sTEA–PCA angle was either a positive or negative value, which meant that cTEA is always externally rotated relative to PCA, but sTEA could be externally or internally rotated.

The sTEA was externally rotated 1.77° to the PCA with a range of 10° which was comparable to the 10° range in the sTEA–PCA angle found by Chalmers et al. [25]. Similarly, several studies have found that the sTEA externally rotated to the PCA by about 2–3° on average [26, 27]. Restrepo et al. also noted that when the posterior cut is made at a fixed 3° external rotation to the posterior condylar axis, there is a significantly greater chance of finding an outlier, which could lead to excessive external or internal rotation of the femoral component, resulting in knee instability and early failure [23].

No consensus exists regarding tibial baseplate positioning during TKA, with two popular techniques used to align the tibial baseplate rotation in TKA. The first utilizes anatomical landmarks, while the second is a range-of-motion (ROM) technique that allows the tibial baseplate to align itself relative to the femoral component by moving the knee through a full range of movement although controversy exists on its reliability [28, 29].

We evaluated the rotational relationship of sTEA with four tibial axes’. i.e., Akagi’s Line, ACA, PTM and the line GC 1/3rd TT, as given in Table 2. From the observations of our study, we found that in a varus knee, the proximal tibia tends to be in an externally rotated position. In our study, we found the Akagi’s line to be externally rotated at a mean of 2.43° with a wide range of 24°; while Aglietti et al. noted it to be on average, approximately 0° [30]. In a meta-analysis, Valkering et al. noted Akagi’s line was reported most frequently and measured 0.7° of internal rotation to the sTEA [29].

Although Lützner et al. found that referencing the tibial rotation on a line from the medial third of the tibial tubercle to the centre of the tibial tray resulted in a better femorotibial rotational alignment than using the medial border of the tibial tubercle as a landmark [31], we found that the line GC 1/3rd TT had the least intra-observer and interobserver reliability with the widest range. Uehara et al. noticed that when the tibial baseplate was aligned to the medial third of the tibial tubercle there was a trend to place the component in external rotation relative to the femoral component [18]. Dalury et al. stated that the tibial baseplate should be rotated externally to the medial 1/3rd of the tibial tubercle to maximize function [20].

Eckhoff et al. also described there was an average of 19° external rotation of the tibial baseplate relative to the femoral component when the tibial tubercle was used as the anatomical landmark and 7° external rotation when PTM was used [7]. They observed a tendency to externally rotate the tibial component relative to the femoral component, which may account for the increased incidence of posteromedial polyethylene wear reported in retrieval studies. In an MRI-based study on 30 normal knees, Incavo et al. reported that, when the alignment of the tibial tray is based upon the posterior tibial condyles, it resulted in an internally rotated component position and only 30% of cases were determined to be in the ideal position [8]. In our study, the mean of the axis recognised to be the closest to sTEA was the PTM and with the highest ICC indicating the best intra-observer reliability, however, interobserver reliability was best seen with ACA.

In our series on the femoral side, the sTEA was not at 3° of external rotation to the PCA in 85% of knees and in 95% the WSL was not perpendicular to sTEA. The PCA had good observer reliability on CT scans. On the tibial measurements, Akagi’s line was more consistent with PTM and ACA having good intra and inter-observer reliability. Our recommendation would be to use sTEA with PCA on the femoral side and Akagi’s line with PTM or ACA on the tibial side with the values depending on individual knee anatomy and soft tissue balance.

The limitations of our study are that it is CT based and may not mirror the intra-operative challenges. Asymmetrical population, with predominantly females in our study. Knees with valgus deformity were not assessed**.** The study was conducted on arthritic knees which could impact the results attributable especially to cartilage erosion, particularly in the posterior condyles**.** Had to include bilateral knee for measurement to get adequate power for this study

## Conclusion

Our findings showed that the “surgical” TEA was not at the assumed 3° of external rotation to PCA in 85% of knees, could be either externally or internally rotated at varying proportions and was neither perpendicular to WSL in >95% of knees. However, the relationship of sTEA with PCA had the best intra-observer and interobserver reliability among the femoral axes. On the tibial side, Akagi’s line had the least variable relationship with sTEA, but PTM had the best intra-observer reliability while ACA had the best interobserver reliability. Though the choice of the above-mentioned rotational reference axes in knee arthroplasty is based on the surgeon’s preference and experience, they should be aware of the multiple femoral and tibial axes that can use in TKA and the range of deviation from sTEA of these reference axes.

## References

[1] Anouchi YS, Whiteside LA, Kaiser AD, Milliano MT (1993) The effects of axial rotational alignment of the femoral component on knee stability and patellar tracking in total knee arthroplasty demonstrated on autopsy specimens. Clin Orthop Relat Res 287, 170–177. 8448937

[2] Berger RA, Crossett LS, Jacobs JJ, Rubash HE (1998) Malrotation causing patellofemoral complications after total knee arthroplasty. Clin Orthop Relat Res 144–153. 10.1097/00003086-199811000-00021 9917679

[3] Nagamine R, White SE, McCarthy DS, Whiteside LA (1995) Effect of rotational malposition of the femoral component on knee stability kinematics after total knee arthroplasty. J Arthroplasty 10, 265–270. 7673902 10.1016/s0883-5403(05)80172-x

[4] Romero J, Stähelin T, Wyss T, Hofmann S (2003) Significance of axial rotation alignment of components of knee prostheses. Orthopade 32, 461–468. 12819884 10.1007/s00132-003-0475-5

[5] Barrack RL, Schrader T, Bertot AJ, Wolfe MW, Myers L (2001) Component rotation and anterior knee pain after total knee arthroplasty. Clin Orthop Relat Res 392, 46–55. 10.1097/00003086-200111000-00006 11716424

[6] Chowdhury EAH, Porter ML (2005) A study of the effect of tibial tray rotation on a specific mobile bearing total knee arthroplasty. J Arthroplasty 20, 793–797. 16139718 10.1016/j.arth.2004.12.058

[7] Eckhoff DG, Metzger RG, Vandewalle MV (1995) Malrotation associated with implant alignment technique in total knee arthroplasty. Clin Orthop Relat Res 321, 28–31. 7497681

[8] Incavo SJ, Coughlin KM, Pappas C, Beynnon BD (2003) Anatomic rotational relationships of the proximal tibia, distal femur, and patella: Implications for rotational alignment in total knee arthroplasty. J Arthroplasty 18, 643–648. 12934219 10.1016/s0883-5403(03)00197-9

[9] Hungerford DS, Krackow KA (1985) Total joint arthroplasty of the knee. Clin Orthop Relat Res 192, 23–33. 3967427

[10] Arima J, Whiteside LA, McCarthy DS, White SE (1995) Femoral rotational alignment, based on the anteroposterior axis, in total knee arthroplasty in a valgus knee: A technical note. J Bone Jt Surg – A 77, 1331–1334. 10.2106/00004623-199509000-00006 7673281

[11] Kobayashi H, Akamatsu Y, Kumagai K, Kusayama Y, Ishigatsubo R, Muramatsu S, et al. (2014) The surgical epicondylar axis is a consistent reference of the distal femur in the coronal and axial planes. Knee Surg Sports Traumatol Arthrosc 22, 2947–2953. 24488236 10.1007/s00167-014-2867-y

[12] Griffin FM, Insall JN, Scuderi GR (1998) The posterior condylar angle in osteoarthritic knees. J Arthroplasty 13, 812–815. 9802670 10.1016/s0883-5403(98)90036-5

[13] Berger RA, Rubash HE, Seel MJ, Thompson WH, Crossett LS (1993) Determining the rotational alignment of the femoral component in total knee arthroplasty using the epicondylar axis. Clin Orthop Relat Res 286, 40–47. 8425366

[14] Yoshino N, Takai S, Ohtsuki Y, Hirasawa Y (2001) Computed tomography measurement of the surgical and clinical trans epicondylar axis of the distal femur in osteoarthritic knees. J Arthroplasty 16, 493–497. 11402414 10.1054/arth.2001.23621

[15] Kobayashi H, Akamatsu Y, Kumagai K, Kusayama Y, Aratake M, Saito T (2015) Is the surgical epicondylar axis the center of rotation in the osteoarthritic knee? J Arthroplasty 30, 479–483. 25466167 10.1016/j.arth.2014.10.024

[16] Thilak J, George MJ (2016) Patient – implant dimension mismatch in total knee arthroplasty: is it worth worrying? An Indian scenario. Indian J Orthop 50, 512–517. 27746494 10.4103/0019-5413.189618 PMC5017173

[17] Akagi M, Oh M, Nonaka T, Tsujimoto H, Asano T, Hamanishi C (2004) An anteroposterior axis of the tibia for total knee arthroplasty. Clin Orthop Relat Res 420, 213–219. 10.1097/00003086-200403000-00030 15057100

[18] Uehara K, Kadoya Y, Kobayashi A, Ohashi H, Yamano Y (2002) Bone anatomy and rotational alignment in total knee arthroplasty. Clin Orthop Relat Res 402, 196–201. 10.1097/00003086-200209000-00018 12218484

[19] Cobb JP, Dixon H, Dandachli W, Iranpour F (2008) The anatomical tibial axis reliable rotational orientation in knee replacement. J Bone Joint Surg 90, 1032–1038. 10.1302/0301-620X.90B8.19905 18669958

[20] Dalury DF (2001) Observations of the proximal tibia in total knee arthroplasty. Clin Orthop Relat Res 389, 150–155. 10.1097/00003086-200108000-00021 11501804

[21] Akagi M, Matsusue Y, Mata T, Asada Y, Horiguchi M, Iida H, Nakamura T (1999) Effect of rotational alignment on patellar tracking in total knee arthroplasty. Clin Orthop Relat Res 366, 155–163. 10.1097/00003086-199909000-00019 10627729

[22] Akagi M, Yamashita E, Nakagawa T, Asano T, Nakamura T (2001) Relationship between frontal knee alignment and reference axes in the distal femur. Clinical Orthop Relat Res 388, 147–156. 10.1097/00003086-200107000-00022 11451114

[23] Restrepo C, Hozack WJ, Orozco F, Parvizi J (2008) Accuracy of femoral rotational alignment in total knee arthroplasty using computer assisted navigation. Comput Aided Surg 13(3), 167–172. 18432416 10.3109/10929080802045640

[24] Daines BK, Dennis DA (2014) Gap balancing vs. measured resection technique in total knee arthroplasty. Clin Orthop Surg 6(1), 1–8. 24605183 10.4055/cios.2014.6.1.1 PMC3942594

[25] Chalmers BP, Kolin DA, Mayman DJ, Miller TM, Jerabek SA, Haas SB, et al. (2021) Three degrees external to the posterior condylar axis has little relevance in femoral component rotation: a computed tomography-based total knee arthroplasty simulation study. J Arthroplasty 36, S380–S385. 33431188 10.1016/j.arth.2020.12.028

[26] Twiggs JG, Dickison DM, Kolos EC, Wilcox CE, Roe JP, Fritsch BA, et al. (2018) Patient variation limits use of fixed references for femoral rotation component alignment in total knee arthroplasty. J Arthroplasty 33, 67–74. 28927560 10.1016/j.arth.2017.08.023

[27] Fitz DW, Johnson DJ, Hartwell MJ, Sullivan R, Keller TJ, Manning DW (2020) Relationship of the posterior condylar line and the transepicondylar axis: A CT-based evaluation. J Knee Surg 33, 673–677. 30959538 10.1055/s-0039-1685146

[28] Saffarini M, Nover L, Tandogan R, Becker R, Moser LB, Hirschmann MT, et al. (2019) The original Akagi line is the most reliable: a systematic review of landmarks for rotational alignment of the tibial component in TKA. Knee Surg Sports Traumatol Arthrosc 27, 1018–1027. 30203197 10.1007/s00167-018-5131-z

[29] Valkering KP, Tuinebreijer WE, Sunnassee Y, van Geenen RCI (2018) Multiple reference axes should be used to improve tibial component rotational alignment: a meta-analysis. J ISAKOS 3, 337–344.

[30] Aglietti P, Sensi L, Cuomo P, Ciardullo A (2008) Rotational position of femoral and tibial components in TKA using the femoral transepicondylar axis. Clin Orthop Relat Res 466(11), 2751–2755. 10.1007/s11999-008-0452-8 PMC2565051 18825470

[31] Lützner J, Krummenauer F, Günther KP, Kirschner S (2010) Rotational alignment of the tibial component in total knee arthroplasty is better at the medial third of tibial tuberosity than at the medial border. BMC Musculoskelet Disord 11, 57. 20338042 10.1186/1471-2474-11-57 PMC2858718

